# Thyroid hormone receptor knockout prevents the loss of *Xenopus* tail regeneration capacity at metamorphic climax

**DOI:** 10.1186/s13578-023-00989-6

**Published:** 2023-02-23

**Authors:** Shouhong Wang, Yuki Shibata, Liezhen Fu, Yuta Tanizaki, Nga Luu, Lingyu Bao, Zhaoyi Peng, Yun-Bo Shi

**Affiliations:** 1 Section on Molecular Morphogenesis, Eunice Kennedy Shriver National Institute of Child Health and Human Development (NICHD), National Institutes of Health (NIH), Bethesda, MD USA; 2 Present Address: Department of Biology, Nippon Medical School, Musashino, Tokyo Japan; 3 Department of Endocrinology, The First Affiliated Hospital of Xi’an Jiaotong University School of Medicine, Xi’an, People’s Republic of China

**Keywords:** Tail regeneration, Metamorphosis, Thyroid hormone, Postembryonic development, *Xenopus laevis*, *Xenopus tropicalis*

## Abstract

**Background:**

Animal regeneration is the natural process of replacing or restoring damaged or missing cells, tissues, organs, and even entire body to full function. Studies in mammals have revealed that many organs lose regenerative ability soon after birth when thyroid hormone (T3) level is high. This suggests that T3 play an important role in organ regeneration. Intriguingly, plasma T3 level peaks during amphibian metamorphosis, which is very similar to postembryonic development in humans. In addition, many organs, such as heart and tail, also lose their regenerative ability during metamorphosis. These make frogs as a good model to address how the organs gradually lose their regenerative ability during development and what roles T3 may play in this. Early tail regeneration studies have been done mainly in the tetraploid *Xenopus laevis* (*X. laevis*), which is difficult for gene knockout studies. Here we use the highly related but diploid anuran *X. tropicalis* to investigate the role of T3 signaling in tail regeneration with gene knockout approaches.

**Results:**

We discovered that *X. tropicalis* tadpoles could regenerate their tail from premetamorphic stages up to the climax stage 59 then lose regenerative capacity as tail resorption begins, just like what observed for *X. laevis*. To test the hypothesis that T3-induced metamorphic program inhibits tail regeneration, we used TR double knockout (TRDKO) tadpoles lacking both TRα and TRβ, the only two receptor genes in vertebrates, for tail regeneration studies. Our results showed that TRs were not necessary for tail regeneration at all stages. However, unlike wild type tadpoles, TRDKO tadpoles retained regenerative capacity at the climax stages 60/61, likely in part by increasing apoptosis at the early regenerative period and enhancing subsequent cell proliferation. In addition, TRDKO animals had higher levels of amputation-induced expression of many genes implicated to be important for tail regeneration, compared to the non-regenerative wild type tadpoles at stage 61. Finally, the high level of apoptosis in the remaining uncut portion of the tail as wild type tadpoles undergo tail resorption after stage 61 appeared to also contribute to the loss of regenerative ability.

**Conclusions:**

Our findings for the first time revealed an evolutionary conservation in the loss of tail regeneration capacity at metamorphic climax between *X. laevis* and *X. tropicalis*. Our studies with molecular and genetic approaches demonstrated that TR-mediated, T3-induced gene regulation program is responsible not only for tail resorption but also for the loss of tail regeneration capacity. Further studies by using the model should uncover how T3 modulates the regenerative outcome and offer potential new avenues for regenerative medicines toward human patients.

**Supplementary Information:**

The online version contains supplementary material available at 10.1186/s13578-023-00989-6.

## Introduction

In adult mammals, most organs/tissues have extremely limited regenerative capacity and only a small number of organs/tissues, such as liver and distal digits, are capable of regeneration upon damage [1–4]. On the other hand, lower vertebrates such as fish, and amphibians have much greater capabilities to regenerate multiple tissues, including heart, tail and limb [5–9]. These organisms with highly regenerative capacity offer great opportunities to determine the mechanisms leading to regenerative failure in higher vertebrate and ultimately provide therapies for human.

Notably, studies on organ/tissue regeneration have been broadly focused on lower vertebrate species (e.g., zebrafish, urodele) with high regenerative abilities and mice with limited regenerative abilities [7, 10–15]. Anurans such as *Xenopus laevis* (*X. laevis*) and *Xenopus tropicalis* (*X. tropicalis*) fill the gap between fish/urodeles and mice for understanding the evolutionary transition in regenerative abilities [9, 16]. In addition, *X. laevis* has been considered as a powerful model to investigate the mechanisms that underlie the changes of organs from being regenerative to non-regenerative during the lifetime of one organism.

Multiple *Xenopus* organs, including, tail, limb, and heart, exhibit stage-dependent regeneration during development, gradually losing the regenerative ability during metamorphosis, which is controlled by thyroid hormone (T3) via T3 receptor (TR)-mediated transcriptional regulation of target genes [16–21]. This suggests that T3 may play an important role in regulating organ/tissue regeneration during *Xenopus* development. Similarly, some human organs, such as the heart, also lose their regenerative ability soon after birth when T3 level is high, but it is difficult to study mammalian postembryonic development as the neonates depend on maternal supply for development and survival [22]. Thus, anurans like *Xenopus* can serve as a model to investigate how organs gradually lose their regenerative ability during development and what roles T3 plays during regeneration.

In addition, *Xenopus* tail can serve as an excellent model to explore the mechanism of regeneration for several reasons. First, the tail consists of many axial and paraxial tissues that are also present in human organs, making it useful for studying regeneration mechanisms for many tissues such as muscle, spinal cord, and vein. Second, it has remarkable regenerative abilities and completes regenerative process fast, within 2 weeks following amputation. Third, it has both regenerative and non-regenerative period during development [8, 23, 24], offering an opportunity to study how regeneration is regulated during development. Previous studies have shown that tail regeneration proceeds through three essential periods: the formation of specialized wound epidermis, blastema bud formation and subsequent patterning and outgrowth via cell proliferation [9, 25–27]. A number of genes/pathways and cellular activities have been found to be involved during these different periods, including reactive oxygen species (ROS), apoptosis, leptin, matrix metalloproteinases (MMPs), Wnt/FGF pathways, mTOR, and cellular metabolism [24–26, 28–32].

Previous studies have observed that *X. laevis* tadpoles can regenerate their tail from larval stages to premetamorphic stages and then gradually lose the regenerative capacity at metamorphic climax [17]. However, only a limited number of stages during metamorphosis were used [9], making is unclear when exactly the tadpole loses the ability to regenerate its tail during metamorphosis. Here, we first compared tail regeneration in both *X. laevis* and *X. tropicalis* at various stages during metamorphosis and found that both *X. laevis* and *X. tropicalis* tadpoles lost their ability for tail regeneration at the climax stages 60/61, just before rapid tail resorption taking place at stage 62 (as reflected by the reduction in tail length), suggesting that T3-induced metamorphic program inhibits tail regeneration. By using wild type and TR double knockout (TRDKO) *X. tropicalis* tadpoles for tail regeneration studies, we found surprisingly that TRs are not necessary for tail regeneration throughout development. Instead, removing both TRs enabled TRDKO tadpoles to retain tail regenerative capacity at the climax stages 60/61. Our analyses suggest that TR-mediated, T3-induced gene regulation program is responsible not only for tail resorption but also for the loss of tail regeneration capacity at metamorphosis climax.

## Results

### TRs are not requited for tail regeneration during *Xenopus* development

**Fig. 1:**
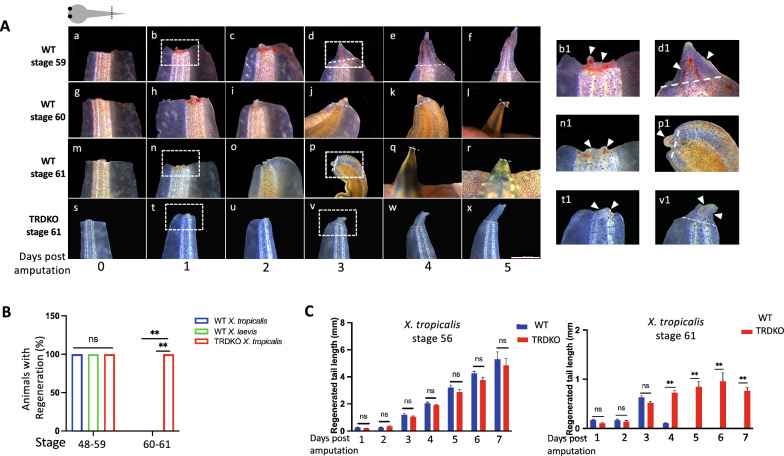
Knocking out both TRα and TRβ (TRDKO) enable tail regeneration at metamorphic climax stage 61 in *Xenopus tropicalis*. **A** Morphological changes during tail regeneration show that tail is able to fully regenerate up to stage 60 but fails to complete the process at the climax stage 61 of metamorphosis in wild type tadpoles while TRDKO tadpoles retain ability to regenerate the tail completely even at stage 61. The white dash lines and white arrowheads indicate amputation site and regenerated portion of the tail, respectively. Right panels are regions in white dashed boxes in left panel at a higher magnification. Scale bar (shown only in Panel x): 1 mm. **B** Percent of animals have tail regeneration at indicated stages 7 days post-amputation. Note that 100% tadpoles of both wild type *X. tropicalis* and *X. laevis*, and TRDKO could regenerate the tail when amputated at all stages between 48 and 59, including stage 48–49, stage 51, stage 54, stage 56, stage 58 and stage 59. At stage 61, 100% of the TRDKO tadpoles could regenerate but none of wild type *X. tropicalis* and *X. laevis* animals had significant regenerated tail 7 days after amputation at stages 60–61. The data were shown as mean values of at least 3 replicates with SE. **P < 0.01, ns: no significant. **C** Quantitative analysis of the length of the regenerated tail reveals that both wild type and TRDKO can regenerate the tail at early metamorphic stage 56 while wild type tail fails to complete tail regeneration at stage 61, unlike the TRDKO tail at stage 61. The length of the regenerated portion of the tail was measured from at least 3 tadpoles at stage 56 or 61 and presented as mean ± SE, ns, no significant

Before investigating the role of TR in tail regeneration by using TR knockout tadpoles, we determined if tail regeneration is similar between *X. tropicalis* and *X. laevis*. Early studies have shown that there is a nutrition dependent divergence between the two species in tail regeneration around the tadpole feeding stage (stage 45), the so-called tail regeneration refractory period in *Xenopus laevis* [27, 31]. On the other hand, when we analyzed tail regeneration after the refractory period, between stage 48 and stage 59 (an early metamorphic stage), we observed that the tail had a robust regenerative ability and completed the entire regenerative process within 1 week after amputation between premetamorphic stage 48 and early metamorphic climax stage 59 in both *X. laevis* and *X. tropicalis* (Fig. 1A, Additional file 1: Fig. S1, and data not shown). For example, at stage 59, *X. tropicalis* tail was able to complete wound healing and form the special wound epidermis at the amputation site around 1 day post-amputation (Fig. 1A). By 3 days post-amputation, regeneration bud was well formed (Fig. 1A), and regeneration was nearly complete 5 days post-amputation (Fig. 1A). Stage 59 *X. laevis* tadpoles behaved similarly, although taking 7 days to fully regenerate (likely due to the lower rearing temperature for *X. laevis* tadpoles (Additional file 1: Fig. S1A). On the other hand, at stages 60 and 61, both *X. laevis* (Additional file 1: Fig. S1) and *X. tropicalis* (Fig. 1) tadpoles failed to regenerate the tail 7 days and 5 days, respectively, after amputation. Interestingly, we observed that in *X. tropicalis*, the tail was able to complete wound healing and form the wound epidermis 1 day after amputation at stages 60/61 (Fig. 1A) and form a regeneration bud by 2–3 days post-amputation (Fig. 1A). However, the subsequent patterning and outgrowth of the regeneration bud were blocked or inhibited and even the limited regeneration observed around 3–4 days post-amputation was resorbed subsequently, suggesting that T3 may also induce resorption of the regenerated tissue at such stages (Fig. 1A). A similar observation was made for *X. laevis* tadpoles at stage 61 (Additional file 1: Fig. S1).

To investigate if TRs affect tail regeneration, we examined tail regeneration in TRDKO *X. tropicalis* animals that lacked any functional TR. The results showed that TRDKO could also fully regenerate the tail after amputation at all stages analyzed, from stage 48 to stage 59 (Fig. 1B, C, and data not shown). To quantify the regenerative capacities of wild type and TRDKO tadpoles, we determined the percent of animals that could regenerate the tail 7 days after amputation in wild type *X. laevis* and *X. tropicalis* tadpoles as well as TRDKO *X. tropicalis* tadpoles according to [27] and found that 100% of the animals of both species or genotypes could regenerate the tail when amputated at stages 48–59 (Fig. 1B). In addition, we measured the length of the regenerated tail at different time points after amputation and found no significant difference between two genotypes at stage 56 at each time point (Fig. 1C). These findings suggest that tail regeneration can occur from premetamorphic stages up to the early metamorphic climax stage 59 in both *X. laevis* and *X. tropicalis* and that TRs are not necessary for tail regeneration in *X. tropicalis.*

### TRDKO enables tail regeneration at the climax stages 60–61

We have shown previously that TRDKO *X. tropicalis* tadpoles are developmentally stalled for up to 2 weeks at around stage 61 before eventual death, whereas wild type tadpoles at stage 61 can complete tail resorption within a week [33–35]. This suggests that TRDKO blocks the tail degeneration program, which may in turn enable tail regeneration at stage 61. To test this, we carried out tail regeneration studies on TRDKO tadpoles at stage 61 and observed that TRDKO tadpoles had essentially complete tail regeneration after amputation at stage 61 (Fig. 1A). Furthermore, 100% of the TRDKO tadpoles could regenerate the tail at stage 61 compared to 0% of wild type stage 61 tadpoles of *X. tropicalis and X. laevis* (Fig. 1B). Additionally, quantification of the length of the regenerated tail during the regeneration period showed that wild type and TRDKO tadpoles amputated at stage 61 had a similarly length of the regenerated tail during the first 3 days post-amputation (Fig. 1C). However, by 4 days post-amputation, the regenerated tail of the wild type tadpoles was resorbed significantly and was not measurable subsequently (Fig. 1C). In contrast, the length of the regenerated tail of the TRDKO tadpoles continued to grow or remain steady after 4 days post-amputation, with a morphology of essentially complete regeneration by 7 days post-amputation (Fig. 1C).

**Fig. 2:**
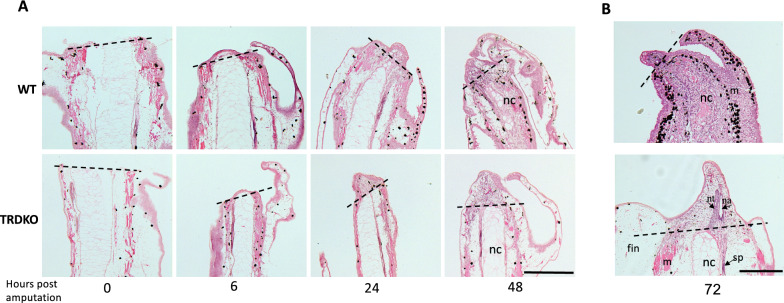
Wild type tadpoles at stage 61 can initiate regeneration after amputation but fails to complete the process. Sagittal sections of tail at different time points after amputation at stage 61 were stained with hematoxylin and eosin. Note that both wild type and TRDKO tail could finish wound healing, form wound epidermis and blastema at 6 h, 24 h and 48 h after amputation, respectively (**A**). At 72 h post-amputation, the tail bud patterning was absent in wild type tadpole, while TRDKO animal had normal regenerated structures, e.g., patterning to form notochord tip and apical ampulla (the termini of the spinal cord) (**B**). nc, notochord; m, muscle; sp, spinal cord; nt, notochord tip; na, neural ampulla. The black dash lines indicate amputation site. Scale bar: 300 μm

Tail regeneration involves three essential periods: the formation of specialized wound epidermis, blastema bud formation and subsequent patterning and outgrowth via cell proliferation. To investigate which periods are responsible for the inhibition of tail regeneration by the T3-induced program during metamorphosis in the wild type animals, histological analyses were carried out at different time point after tail amputation of stage 61 wild type and TRDKO tadpoles. The results revealed that both wild type and TRDKO tail could initiate regeneration, with complete wound healing and formation of the special wound epidermis and blastema by 48 h post-amputation (Fig. 2A, Additional file 1: Fig. S2). Subsequently, the wild type and TRDKO tadpoles diverged. In the wild type animals, there was a loss of regenerative tissue during patterning and outgrowth period by 72 h or 3 days post-amputation (Fig. 2B). On the other hand, patterning and outgrowth continued between 48 and 72 h post-amputation in the TRDKO tadpoles (Fig. 2B). In fact, by 72 h post-amputation, notochord precursor cells accumulated to create a compact cell mass (notochord tip) adjacent to the edge of the amputated notochord sheath and the spinal cord to form a neural ampulla in the regenerating part of tail in TRDKO but not in wild type tadpoles (Fig. 2B). Thus, removing TRs prevents the loss of tail regenerative capacity at the climax of metamorphosis.

### TRDKO increases amputation-induced apoptosis in the earlier period of regeneration as well as subsequent cell proliferation in outgrowth period

**Fig. 3:**
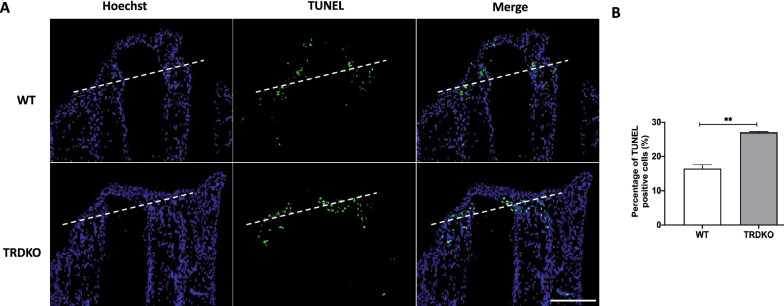
TRDKO tadpoles have more amputation-induced apoptosis at 6 h post-amputation at stage 61. **A** TUNEL labeling (green) was carried out on sagittal sections of the tail at 6 h post-amputation, counterstained with Hoechst 33342 (blue), to detect apoptotic cells. Note that apoptotic cells appeared around the amputation site in both wild type and TRDKO. The white dash lines indicate amputation site. Scale bar: 300 μm. **B** Quantification of apoptotic cells. The TUNEL positive cells (green) were counted with ImageJ software and normalized against the total Hoechst positive cells (blue). The data are presented as mean ± SE (n = 3). **P < 0.01

**Fig. 4:**
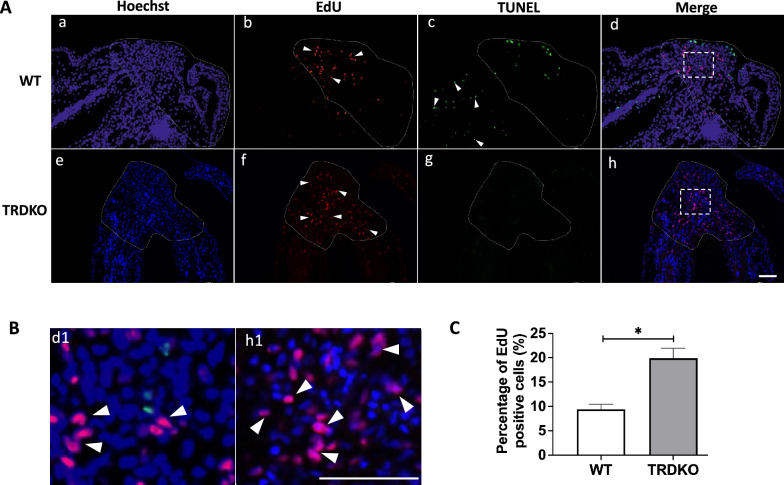
TRDKO tadpoles have more cell proliferation in the regenerated portion of the tail with no or fewer apoptotic cells in the uncut portion of the tail at 48 h post-amputation at stage 61 compared to the wild type animals. **A** EdU (red) labeling of proliferating cells was carried out on sagittal sections of tail at 48 h post-amputation, counterstained with TUNEL (green) for apoptotic cells and Hoechst 33342 (blue) for DNA, respectively. White arrow heads point to representative labeled cells. The regenerated portion of the tail is encircled with dotted lines. Scale bar: 50 μm. **B** A higher magnification of area in the white dashed boxes in **A.** White arrow heads point to representative labeled proliferating cells. Scale bar: 50 μm. **C** Quantification of proliferating cells in the regenerated portion of the tail (areas encircled in **A**) of both wild type and TRDKO tadpoles. The EdU positive cells (red) were counted with ImageJ software and normalized against the total Hoechst positive cells (blue) and presented as mean ± SE (n = 3), *P < 0.05

**Fig. 5:**
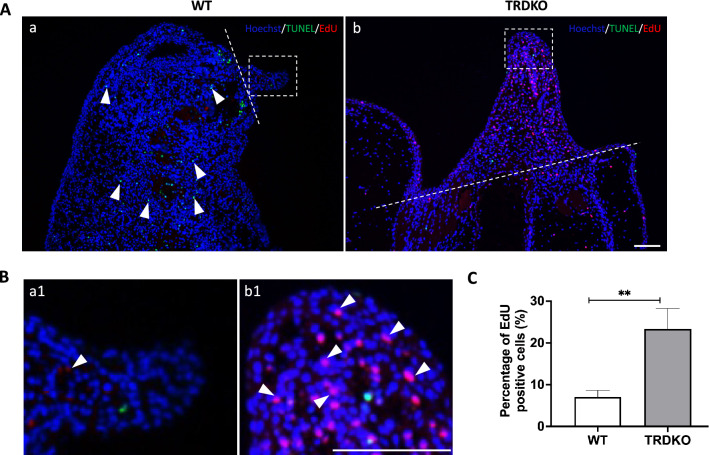
The wild type animals lose cell proliferation in the regenerating region at 72 h post-amputation while the regenerating tail of the TRDKO tadpoles continue to have high levels of cell proliferation. **A** EdU (red) labeling of proliferating cells was carried out on sagittal sections of tail at 72 h post-amputation, counterstained with TUNEL (green) for apoptotic cells and Hoechst 3342 (blue) for DNA, respectively. The white dash lines indicate amputation sites and the white arrowheads point to labeled cells. Scale bar: 100 μm. **B** A higher magnification of area in the white dashed boxes in **A**. White arrow heads point to labeled proliferating cells. Scale bar: 100 μm. **C** Quantification of proliferating cells in the regenerated portion of the tail of wild type and TRDKO tadpoles. The EdU positive cells (red) were counted with ImageJ software and normalized against the total Hoechst positive cells (blue) and presented as mean ± SE (n = 3), **P < 0.01

To assess how wild type tail failed to complete the regeneration after amputation at stage 61, we first investigated the apoptosis and cell proliferation after amputation in both wild type and TRDKO tadpoles given that these processes are indispensable for tail regeneration during the first (formation of specialized wound epidermis) and third (patterning and outgrowth) period, respectively [29, 31]. Using TUNEL staining of the sagittal section at 6 h post-amputation, we found that the majority of apoptotic cells were near the amputation site in both wild type and TRDKO tail (Fig. 3A). Quantitative analysis showed that the number of apoptotic cells was significantly higher in TRDKO tail compared to that in wild type tail (Fig. 3B). In addition, RT-qPCR analysis showed that the expression of 3 apoptotic genes, *caspase 9*, *bax* and *fas,* in the regenerated portion of tail was significantly induced at 6 h after amputation in both wild type and TRDKO tail (Additional file 1: Fig. S3A, B), and two of them had slightly higher folds of induction in TRDKO tail (Additional file 1: Fig. S3C), consistent with higher levels of apoptosis in the TRDKO animals. Similarly, EdU-labeling proliferating cells [36] at 48 (Fig. 4) and 72 h (Fig. 5) post-amputation showed that TRDKO tadpoles had significantly more proliferating cells in newly regenerated portion of the tail compared to that in wild type tadpoles. Interestingly, proliferating cells relative to the total cell number in the newly regenerated portion of the tail actually increased between 48 and 72 h post-amputation in TRDKO tadpoles, but decreased in wild type tadpoles (compared Figs. 5C to 4C). When we analyzed the expression of two cell cycle genes, *cdk1* and *cdca8,* at 0, 24 and 72 h after amputation, we found that they were upregulated by 24 h in both wild type and TRDKO (Additional file 1: Fig. S4). However, in wild type animals, their expression then decreased by 72 h while in TRDKO animals, the expression increased further (Additional file 1: Fig. S4), again consistent with the cell proliferation data. Furthermore, there were abundant apoptotic cells in the uncut portion of the tail of wild type tadpoles at 48 and 72 h post-amputation due to natural tail resorption as the tadpole developed further from stage 61 (Figs. 4Ac, 5Aa). In contrast, such apoptotic cells were absent in the uncut portion of the tail of TRDKO tadpoles as TRDKO inhibited tail resorption (Figs. 4Ag, 5Ab). These results are consistent with our morphological observations and histology data and suggest that the increased apoptosis at early regenerative period and subsequent cell proliferation after amputation in the TRDKO tadpoles help to enable tail regeneration at stage 61. They further argue that T3-induced, TR-mediated tail resorption program inhibits tail regeneration.

### TRDKO enhances amputation-induced expression genes known to be involved in regeneration at the climax of metamorphosis

**Fig. 6:**
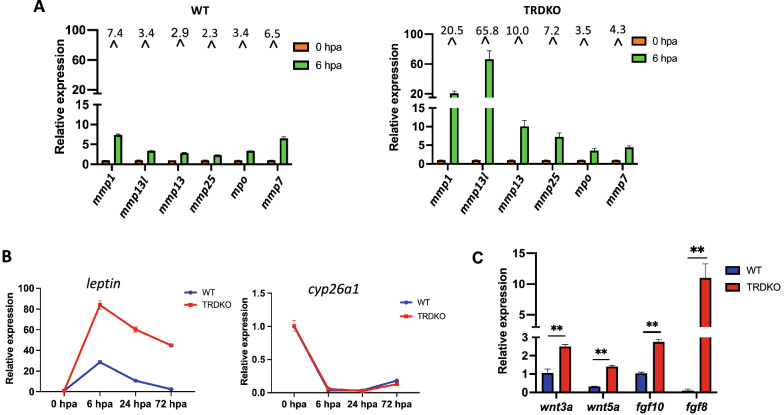
At the non-regenerative stage 61, wild type tadpoles have reduced upregulation of genes known to be induced during tail regeneration compared to TRDKO tadpoles. **A** The expression of reparative myeloid genes at 0 h and 6 h after amputation in wild type tail and TRDKO tail. The expression was determined by RT-qPCR and normalized to that of *rpl8*. Note that all genes: *mmp1* (matrix metalloproteinases 1), *mmp13l*, *mmp25, mpo* (myeloperoxidase), and *mmp7*, were upregulated during early stage of regeneration in both wild type and TRDKO tail after amputation but TRDKO enhanced the induction of *mmp1*, *mmp13l*, *mmp13*, *mmp25* compared to wild type tadpoles. **B** TRDKO enhances the upregulation of *leptin* but not *cyp26a1* during tail regeneration after amputation at stage 61. Notably, *leptin* has reported the highest significant upregulation in the 6 h vs 0 h comparison while *cyp26a* possesses the greatest significant decreased in the 6 h vs 0 h comparison during tail regeneration in *Xenopus*. **C** TRDKO leads to higher levels of expression of Wnt and FGF genes during the patterning and outgrowth period of tail regeneration, 72 h post-amputation at stage 61. All gene expression data were presented as mean ± SE, **P < 0.01

We next investigated whether TRDKO affects the expression of genes known to be involved in tail regeneration by using RT-qPCR analyses of tail tissue at different time points after amputation at stage 61. We first analyzed three inflammatory genes, *il1b*, *il8* and *il1r2*, known to be regulated during regeneration, and found that all were upregulated by 6 h after amputation in both wild type and TRDKO tail (Additional file 1: Fig. S5). We then analyzed 6 known reparative myeloid genes: matrix metalloproteinase 1 (*mmp1*), mmp7, *mmp13*, *mmp13l*, *mmp25*, and *mpo* (myeloperoxidase) [30]. All of them were found to be upregulated at 6 h post-amputation in both wild type and TRDKO tail. However, the fold induction by amputation was much higher in the TRDKO tadpoles for 4 of the 6 genes, *mmp1* (20.5-fold vs 7.4-fold), *mmp13l* (65.8-fold vs 3.4-fold), *mmp13* (10.0-fold vs 2.9-fold) and *mmp25* (7.2-fold vs 2.3-fold), compared to that in wild type tadpoles (Fig. 6A). We next assessed the expression of 2 genes known to be among the most significantly regulated genes after tail amputation and likely involved in the regeneration of different tissues/organs, suggesting that they are the conserved markers that be more useful for our experiment: the upregulated gene *leptin* and downregulated gene *cyp26a* [24]. We found that *leptin* was induced to peak level at 6 h post-amputation and the expression level then dropped to lower levels at 24 and 72 h in both wild type and TRDKO tail (Fig. 6B). Here again, TRDKO led to a much stronger induction of *leptin* at 6 h post-amputation compared to wild type animals. On the other hand, *cyp26a* was downregulated similar in the TRDKO and wild type tail (Fig. 6B).

Finally, we analyzed the expression of 4 genes likely involved in the patterning and outgrowth of the regenerated tail, including *wnt3a*, *wnt5*a, *fgf10* and *fgf8* [17], at 72 h post-amputation in both wild type and TRDKO tadpoles. We found that the expression levels of Wnt and FGF genes (*wnt3a*, *wnt5*a, *fgf10* and *fgf8*) in wild type tail were significantly lower than those in TRDKO tail at 72 hpa (Fig. 6C). These data indicate that TRDKO enhanced amputation-induction of the expression of many genes important for different periods of tail regeneration to allow the tadpole to retain tail regenerative capacity at the climax of metamorphosis.

## Discussion

Tissue regeneration is critical for organ function and long-term survival of different organisms. The ability for tissue regeneration generally decreases from lower to higher organisms and from immature/neonatal/larval to mature/adult organs, with mammalian adult organs having perhaps the lowest ability to regenerate. Postembryonic development in mammals or the equivalent period, i.e., metamorphosis, in amphibians is accompanied by a drastic reduction of the ability of many organs to regenerate. T3 is known to be critical for postembryonic development in vertebrates and thus may play a role in regulating the regenerative ability of vertebrate organs during development. Here, our study using genetic and developmental approaches is the first to demonstrate that TR is not required for tail regeneration throughout *Xenopus* development but T3-induced, TR-mediated tail resorption program inhibits tail generation. Our findings suggest that T3-induced adult organ development or larval tissue degeneration is responsible for the reduction or loss of organ regenerative ability during development.

Our studies here first provided morphological evidence that the tail gradually loses the regenerative ability at metamorphic climax stages 60–61 in both *X. laevis and X. tropicalis*, similar to that reported for cardiac regeneration in *X. laevis* [16]. In addition, a number of other organs, such as the limb, spinal cord, lens, and brain also lose or reduce their regenerative ability during metamorphosis [37–40]. Interestingly, the loss of regenerative ability in all these organs, including the tail, coincides with the metamorphic changes in these organs, i.e., developing into their adult forms (de novo formation of the limb, resorption of the tail, and remodeling of other organs) during metamorphosis. Since all metamorphic changes are controlled by T3 through transcriptional regulation of target genes by TRs [21, 41–46], T3-signaling through T3 is likely important in regulating the regenerative ability of these organs during development. Indeed, our findings in TRDKO tadpoles revealed that blocking T3 signaling by removing TR at the climax of metamorphosis when tail resorption normally occurs prevented the loss of tail regenerative ability [33].

Interestingly and perhaps surprisingly, TRDKO tadpoles prior to the stages of tail resorption could regenerate the tail. Thus, TRs are themselves not required for tail regeneration. Instead, it is the metamorphic program, which, in the case of the tail, is induced by T3 via TR at the climax of metamorphosis, that inhibits tail regeneration. In this regard, it is worth noting that limb regeneration can also occur in TRDKO tadpoles but is inhibited as limb metamorphosis occurs even in TRDKO tadpoles (data not shown). Since TR functions as repressors of T3-inducible genes as unliganded receptor, e.g., during premetamorphosis when T3 level is low, and as activators of these same genes when liganded, e.g., during metamorphosis when T3 level is high. De-repression of the genes due to TRDKO is sufficient for adult organ development during metamorphosis [33] and thus, it is not be surprising that limbs also lose regenerative ability in TRDKO tadpoles since it can undergo metamorphosis in TRDKO tadpoles. In this regard, it is worth noting that heart regenerative ability is also regulated by T3 during postembryonic development in both *Xenopus* and mouse [16, 47], likely due to T3-dependent maturation of the heart during *Xenopus* metamorphosis or mouse postembryonic development [48]. Thus, it is likely that the development program responsible for the maturation of individual organs/tissues causes the loss of regenerative ability in these organs/tissues.

At cellular and molecular levels, the regeneration of TRDKO tail after amputation at the climax of metamorphosis resembles that of wild type tail after amputation during premetamorphosis. Previous studies have shown that early during regeneration, apoptosis plays an important role in determining the regenerative ability in many organisms by facilitating the release of signaling molecules, such as Wnt and prostaglandin E2, etc., and affects subsequent cell proliferation and tissue patterning [26, 29, 49–51]. Our results indicate that wild type tadpoles at climax stage 61 can initiate the regenerative process but with a lower level of apoptosis, suggesting the reduced or incomplete imitation of regeneration compared to TRDKO tadpoles at climax stage 61. It has been reported that the induction of apoptosis after injury requires mitogen-activated protein kinase (MAPK) pathway [52–54]. Interestingly, we have observed that genes downregulated in the tail by T3 treatment of wild type tadpoles are significantly enriched with genes in MAPK pathway, while such an enrichment is not observed for TRDKO tadpoles [55] suggesting that T3 may regulate MAPK pathway via TR to affect tail regeneration. In addition, we observed that most of the analyzed reparative myeloid genes were induced at much lower levels in the wild type tail compared to TRDKO tail 6 h after amputation at stage 61 (Fig. 6A). The myeloid lineage activities have been shown to be upstream of apoptosis and can affect the apoptosis during regeneration [30]. Our findings thus suggest that T3-signaling may affect myeloid lineage activity to regulate amputation-induced apoptosis during the early period of tail regeneration, consistent with earlier reports that T3 targets macrophages to affect the pro-inflammatory nuclear factor-κB activities, which is required for successful regeneration [56–59].

In addition, organ/tissue regeneration shares certain hallmarks of embryonic development, especially during the patterning and outgrowth period, e.g., both requiring rapid cell proliferation [60–62]. Our results show that the number of proliferating cells was significantly lower, especially during the patterning and outgrowth period, in the regenerating tail of wild type tadpoles compared with TRDKO tadpoles after amputation at stage 61. In addition, histological analyses showed that the regenerating tail in the TRDKO tadpoles at stage 61 had normal patterning, including the formation of notochord tip, by 72 h post-amputation, whereas little patterning was observed for the wild type tadpoles. The wild type, but not TRDKO, tadpole tail, however, had high levels of apoptosis in the uncut portion of the tail at 48 h and 72 h post-amputation at stage 61 due to the activation of tail resorption program after stage 61. Thus, cell–cell interaction and dynamic cellular behaviors may be critical for patterning during regeneration by providing a regeneration-permissive environment [63].

**Fig.7:**
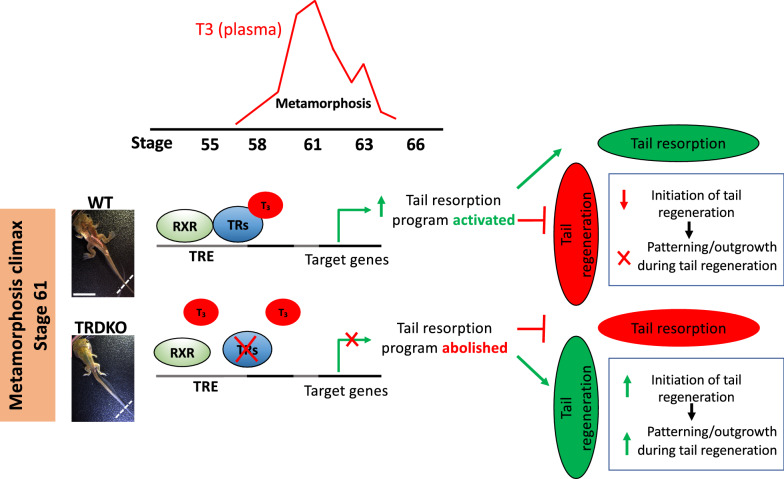
A model for tail regeneration at stage 61 by T3. In wild type animals at metamorphic climax stage 61, T3 peaks and liganded T3 receptors (TRs) recruit coactivator complexes to activate gene transcription responsible for tail resorption and inhibit tail regeneration, including inhibition of the initiation of the regeneration after amputation and preventing subsequent patterning and outgrowth. In TRDKO tadpoles, the activation of these genes by T3 is absent, thus preventing tail resorption and allowing the tail to retain regenerative ability. RXR, 9-cis-retinoic acid receptor. TRE, T3 response element. The white dash lines indicate amputation sites. Scale bar: 8.7 mm

A number of studies have analyzed the gene expression changes during tail regeneration, development, and resorption [35, 62, 64–66]. A number of genes have been found to be strongly upregulated after tail amputation. One of them, *leptin*, is upregulated early after amputation to promote organ/tissue regeneration in different animal species [24, 67–69]. Its upregulation was much lower in the wild type tail compared to TRDKO tail at 6 h after amputation at stage 61. Notably, leptin is a nutritionally regulated hormone, and its protein was found to be localized in the wound epidermis and it could activate both JAK/STAT3 and MAPK/ERK signaling to affect the regenerative outcome in limb regeneration in *Xenopus laevis* [70]. In addition, previous study has showed that nutrient state can affect tail regeneration in *X. tropicalis* [31], suggesting that T3/TRs may affect tail regeneration at least in part through leptin signaling. Another gene of interest, *cyp26a1,* which is involved in retinoic acid (RA) clearance and is among the most dramatic down-regulated genes during the wound healing during normal tail regeneration in *Xenopus tropicalis* [24,] suggested this gene regulation is important at least during the initiate stage of *Xenopus* tail regeneration. We showed that in both wild type and TRDKO tadpoles at stage 61, the gene was regulated in a similar regulation pattern, further supporting our conclusion that wild-type tail could initiate the tail regeneration just like the TRDKO tail at stage 61. Furthermore, RA signaling, through RAR (RA receptor)-RXR heterodimers can influence the formation, proliferation, and survival of the blastema during adult zebrafish fin regeneration [71]. This suggests that the regulation of *cyp26a1* may related to the RA signaling during tissue/organ regeneration. It would be interesting to investigate whether RXR-RAR heterodimers play a role in tail regeneration in TRDKO. In addition, Wnt and FGF pathways are well-known signaling pathways implicated in tail and limb regeneration [26, 72, 73]. We found that *wnt3a*, *wnt5a*, *fgf10* and *fgf8* were expressed at much higher levels in the patterning and outgrowth period in the TRDKO tail compared to wild type tail after amputation at stage 61. Our data are consistent with the observation that Wnt and FGF are required for spinal cord and muscle regeneration in *Xenopus* tail regeneration [74, 75], suggesting that T3 regulated genes through TRs may affect the outgrowth of the regenerating tail through Wnt and FGF signaling pathways. Altogether, our morphological, histological, and molecular findings support a model where T3 regulates gene expression programs through TRs to induce tail resorption and the tail resorption program in turn contribute to the inhibition of the initiation as well as the patterning and outgrowth period of tail regeneration (Fig. 7).

## Conclusions

Our studies here for the first time reveal that developmental regulation of tail regeneration is conserved between *X. laevis* and *X. tropicalis* except during the refractory period, around the onset of feeding when *X. laevis* cannot but *X. tropicalis* can regenerate its tail after amputation [27]. By using total TR knockout in the diploid *X. tropicalis,* we have discovered that while TR is not needed for tail regeneration, TR-mediated, T3-induced tail resorption program might be responsible for the loss of regenerative ability at/after climax stages 60/61. Given that other organs/tissues, such as the heart [16], also lose/decrease their ability to regeneration during T3-dependent metamorphosis, it is likely that T3-dependent maturation of adult organs/tissues or resorption of the larval organs inhibits tissue/organ regeneration. This raises the possibility of manipulating hormone signaling to revert tissues/organs to their neonatal/larval state, such as TRDKO for tadpole tail, toward developing regenerative medicines for human diseases.

## Materials and methods

### Experimental animals

Wild type *X. laevis and X. tropicalis* adults were purchased from Nasco or raised in the laboratory. Tadpoles were staged according to [76]. Sexually mature *X. tropicalis* frogs homozygous for TRα knockout and heterozygous for TRβ knockout [(TRα (−/−)β(±)] were mated to produce TRDKO [(TRα (−/−)β(−/−)] tadpoles [33]. *X. laevis* and *X. tropicalis* embryos/tadpoles were maintained at 25 °C and 20 °C, respectively. All animal care and treatments were performed as approved by the Animal Use and Care Committee of *Eunice Kennedy Shriver* National Institute of Child Health and Human Development of the National Institutes of Health.

### Genotyping

Tadpoles were anesthetized with 0.02% MS222 (TCI, Tokyo, Japan) and tadpole tail tip (about 5 mm or less) was clipped and lysed in 20 μL QuickExtract DNA extraction solution (EPICENTRE Biotechnologies, Madison, WI, USA) at 65 °C for 20 min. After incubation at 95 °C for 2 min and centrifugation at 4000 rpm for 10 min, 1 μL of the resulting DNA solution was immediately used for genotyping [43]. Briefly, the genotyping for TRβ wild type or the 19-base deletion mutant was done by PCR with forward primer, 5′-GGACAACATTAGATCTTTCTTTCTTTG-3′ and reverse primer, 5′-CACACCACGCATAGCTCATC-3′ for 33 cycles of 94 °C for 30 s, 60 °C for 30 s, and 72 °C for 20 s. The PCR products were analyzed by 3.5% agarose gel electrophoresis to determine the genotype based on the sizes of the products.

### EdU labeling

To detect proliferating cells, 10 mg/mL 5-ethynyl-2′-deoxyuridine (EdU) was injected into tadpoles as previously reported [36]. Briefly, tadpoles at the indicated stages were anesthetized in 0.02% MS222 until they stop moving. Tadpoles at stage 61 were placed on a paper towel and injected intraperitoneally with 10 μL of 10 mg/mL EdU using a Hamilton syringe into the abdominal cavity. 30 min after EdU injection, tadpoles were euthanized, and tissue were harvested.

### Amputation procedure

Tadpoles at the indicated stages were first anesthetized in 0.02% MS222 in 0.1X MMR [0.1 M NaCl, 2.0 mM KCl, 1 mM MgSO4, 2 mM CaCl2, 5 mM HEPES (pH 7.8)]. They were transferred to fresh 0.1X MMR before amputation to remove 30–50%, which ever was less, of the tail by using a sterile scalpel. The amputated tadpoles were kept in a tank containing 0.1X MMR and 50 μg/ml gentamicin for the first 2 days. The amputated *X. laevis* and *X. tropicalis* tadpoles were kept in 20 °C and 25 °C incubators, respectively, for 7 days with daily change of 50% rearing water without gentamicin starting from day 3. The percent of animals with regeneration at stage 61 was assessed at 7 days post-amputation with regeneration considered to be good or excellent as previously described [27].

### Histological study

Tissues were fixed with 4% paraformaldehyde overnight, stored in 70% ethanol for up to 2 weeks, embedded in paraffin by using a tissue processor, and sectioned at 5 μm. After deparaffinization, the tissue sections were stained with hematoxylin and eosin (H&E) staining following the manufacturer’s protocol (Sigma-Aldrich) and analyzed under a bright-field microscope.

### TUNEL assay and EdU staining

Apoptotic cells were detected by using terminal deoxynucleotidyl transferase-mediated dUTP nick-end labeling (TUNEL) with fluorescein in situ cell death detection kit (#11684795910, Roche). Briefly, 5 µm paraffin sections were baked at 60 °C for 30 min followed by deparaffinization with xylene and rehydrated through a graded series of ethanol. Antigen retrieval was performed by microwaving the sections (700 W; 2 min) in sodium citrate buffer (pH 6.0) followed by rinsing in PBS. The sections were incubated for 30 min in 0.1 M Tris–HCL (pH 7.5) containing 1.5% bovine serum albumin and 20% normal bovine serum at room temperature to block non-specific binding sites, washed in PBS, and incubated with TUNEL reaction mixture at 37 °C for 1 h. After removing the TUNEL reaction mixture, sections were washed in PBS 3 times and followed by detecting proliferating cells with the Click-iT Plus EdU Alexa Fluor 594 Imaging kit (#C10339, Invitrogen). Briefly, sections were incubated with Click-iT® Plus reaction cocktail for 30 min at room temperature, washed 3 times with TBST (1× TBS and 0.05% Tween-20), and then counter-stained with Hoechst 33342 (1:2000) for 30 min at room temperature before washing 3 times with TBST. They were then mounted on glass slides with ProLong™ Gold antifade regent (#P36930, Thermo Fisher Scientifc). The fluorescent pictures for different colors and different sections were taken under the same settings and then analyzed with ImageJ software (National Institutes of Health). Briefly, after opening the fluorescent images in ImageJ, all images were changed from RGB color to 8-bit format and Image-Adjust-Threshold-Apply was used to adjust the images. Next, by using watershed to separate the compacted cells by choosing process binary-watershed. Finally, Analyze-Analyze Particles was selected to count the cell number automatically. Both the percentage of TUNEL- and EdU-positive cells were obtained by normalizing against the total Hoechst 33342 positive cells.

### Quantitative reverse-transcription PCR (RT-qPCR)

At 0 h, 6 h, 24 h and 72 h post-amputation of the tail of wild type and TRDKO tadpoles at stage 61, the part of the tail including all regenerated tail plus about 250 μm of the original uncut tail proximal to the site of amputation was dissected for total RNA isolation with RNeasy^®^ Mini Kit 250 (QIAGEN). Reverse transcription on the RNA was carried out with a High-Capacity cDNA Reverse Transcription kit (Applied Biosystems, Waltham, MA). Briefly, total RNA (500 ng) was reversed transcribed into cDNA in a 20 μL reaction including 2 μL of RT Buffer (× 10 concentrate), 2 μL of RT Random Primers (× 10 concentrate), 0.8 μL of dNTP mix (100 mM), and 1 μL of Multiscribe™ Reverse Transcriptase (50 U/μL). The mixture was incubated at 25 °C for 10 min, 37 °C for 120 min, and 85 °C for 5 min. Then, it was diluted 1:10 with Nuclease-free water and 2 μL of the resulting cDNA solution was added to a quantitative PCR mixture containing 10 μL of 2× SYBR Green PCR Master Mix (Applied Biosystems, Foster City, CA), 2 μL of primers (10 μM, 1 μL Forward primer and 1 μL Reverse primer) and 6 μL Nuclease-free water. Quantitative reverse-transcription PCR (RT-qPCR) was performed in triplicates by using Step One Plus Real-Time PCR System (Applied Biosystems). Expression values were calculated by using the ΔΔCt method with *rpl8* used as a control [77], and the deviation was calculated by using standard error of the mean (SEM). Primers are shown in Additional file 1: Table S1.

### Statistical analysis

Data are presented as mean ± SE. The significance of differences between groups was evaluated by Student’s *t*-test by using Prism 9 (GraphPad Software, La Jolla, CA, USA).

## Supplementary Information

**Additional file 1: Figure S1. **Tail loses regenerative ability during metamorphic climax in Xenopus laevis. (A) Representative images of the tail at different time points after amputation for two tadpoles. The tadpole at stage 59, an early metamorphic climax stage, regenerated the tail completely by 7 days (a), while the one stage 61, a later stage when plasma T3 is around the peak level, failed to regenerate (b). Scale bar: 1mm. (B) Quantitative analysis of the length of the regenerated tail after amputation at stage 56, an early metamorphic stage, or at stage 61. Note that regenerated tail gradually increased in length after amputation at stage 56, while the length of the regenerated tail at stage 61 appeared to decrease after 4 days, likely due to tail resorptions. The length of the regenerated portion of the tail was measured from at least 3 tadpoles at stage 56 or 61 and presented as mean ± SE. **P < 0.01, ns, not significant. **Figure S2.** Tail can initiate regeneration in both wild type and TRDKO tadpoles at stage 61. Frontal sections of wild type (a, b) and TRDKO (c, d) tadpole tail at 24 hours post-amputation (hpa) and 48 hpa that were stained with hematoxylin and eosin. Note that both wild type and TRDKO could complete wound healing and form special wound epidermis and blastema (as indicated in black arrowheads). Black dash lines indicate amputation site. nc, notochord; m, muscle. Scale bar: 150 μm. **Figure S3. **Analysis of the expression of apoptotic genes during wound healing in both wild-type (WT) and TRDKO animals by RT-qPCR. The expression of three apoptotic genes (*caspase 9*, *bax, *and *fas*) at 0 hr and 6 hr after amputation in wild-type (A) and TRDKO tail (B). Each bar represents the mean plus S.E. and (*) indicates a significant difference between 6 hr and 0 hr (P < 0.05). (C) The ratio of the expression of the same three apoptotic genes at 6 hr to that at 0 hr for WT and TRDKO tail. Each bar represents the mean plus S.E. and (*) indicates a significant difference between the WT and TRDKO tail (P<0.05). ns indicates no significant difference. Note that the genes were induced during wound healing in both WT and TRDKO tadpoles, with TRDKO animals having a higher induction for two of the genes, consistent with the TUNEL staining results. **Figure S4. **The regulation of cell cycle genes at patterning and outgrowth period during tail regeneration in both wild-type (WT) and TRDKO. The expression of two known cell cycle genes (*cdk1* and *cdca8*) was analyzed by RT-PCR at 0 hr, 24 hr and 72 hr after amputation in wild-type (A) and TRDKO tail (B). Each bar represents the mean plus S.E. and (*) indicates a significant difference between 24 hr and 0 hr or 72 hr and 0 hr (P < 0.05). ns indicates no significant difference. Note that both genes were upregulated at 24 hr in both WT and TRDKO tadpoles. However, in WT tadpoles, their expression at 72 hr were returned to lower levels. These were consistent of the EdU staining results. **Figure S5. **Analysis of the expression of inflammatory genes during wound healing in both wild-type (WT) and TRDKO animals by RT-qPCR. The expression of three inflammatory genes (*il1b*, *il8 *and *il1r2*) at 0 hr and 6 hr after amputation in wild-type (A) and TRDKO tail (B). Each bar represents the mean plus S.E. and (*) indicates a significant difference between 6 hr and 0 hr (P < 0.05). Note that the genes were induced during wound healing in both WT and TRDKO tadpoles. **Table S1.** Primers used in RT-qPCR.

## Data Availability

Not applicable.

## Abbreviations

*X.*: *Xenopus*
T3: Thyroid hormone
TRs: Thyroid hormone receptors
TRDKO: TR double knockout (knocking out both TRα and TRβ genes)
hpa: Hours post-amputation
dap: Days post-amputation
ROCs: Regeneration-organizing cells
ROS: Reactive oxygen species
Wnt: Wingless-related integration site
FGF: Fibroblast growth factor
MAPK: Mitogen-activated protein kinase
ERK: Extracellular signal-regulated kinase
JAK: Janus kinase
STAT: Signal transducer and activator of transcription
hCG: Human chorionic gonadotropin
caspase: Cysteine-aspartic proteases
bax: Bcl-2 associated x-protein
il: Interleukin
cdk: Cyclin-dependent kinase
cdca: Cell division cycle associated
RA: Retinoic acid
mmp: Matrix metalloproteinases
EdU: 5-Ethynyl-2-deoxyuridine
TUNEL: Terminal deoxynucleotidyl transferase dUTP nick end labeling
RT-qPCR: Reverse transcription quantitative real-time PCR
RXR: 9-Cis-retinoic acid receptor
TRE: T3 response element
