# Supplementary material for: Thyroid hormone receptor knockout prevents the loss of Xenopus tail regeneration capacity at metamorphic climax
Source: Cell Biosci. 2023 Feb 23;13:40. doi: 10.1186/s13578-023-00989-6 (PMC9948486; doi:10.1186/s13578-023-00989-6)
Supplement: Supplementary file 1 — Additional file 1: Figure S1. Tail loses regenerative ability during metamorphic climax in Xenopus laevis. (A) Representative images of the tail at different time points after amputation for two tadpoles. The tadpole at stage 59, an early metamorphic climax stage, regenerated the tail completely by 7 days (a), while the one stage 61, a later stage when plasma T3 is around the peak level, failed to regenerate (b). Scale bar: 1mm. (B) Quantitative analysis of the length of the regenerated tail after amputation at stage 56, an early metamorphic stage, or at stage 61. Note that regenerated tail gradually increased in length after amputation at stage 56, while the length of the regenerated tail at stage 61 appeared to decrease after 4 days, likely due to tail resorptions. The length of the regenerated portion of the tail was measured from at least 3 tadpoles at stage 56 or 61 and presented as mean ± SE. **P < 0.01, ns, not significant. Figure S2. Tail can initiate regeneration in both wild type and TRDKO tadpoles at stage 61. Frontal sections of wild type (a, b) and TRDKO (c, d) tadpole tail at 24 hours post-amputation (hpa) and 48 hpa that were stained with hematoxylin and eosin. Note that both wild type and TRDKO could complete wound healing and form special wound epidermis and blastema (as indicated in black arrowheads). Black dash lines indicate amputation site. nc, notochord; m, muscle. Scale bar: 150 μm. Figure S3. Analysis of the expression of apoptotic genes during wound healing in both wild-type (WT) and TRDKO animals by RT-qPCR. The expression of three apoptotic genes (caspase 9, bax, and fas) at 0 hr and 6 hr after amputation in wild-type (A) and TRDKO tail (B). Each bar represents the mean plus S.E. and (*) indicates a significant difference between 6 hr and 0 hr (P < 0.05). (C) The ratio of the expression of the same three apoptotic genes at 6 hr to that at 0 hr for WT and TRDKO tail. Each bar represents the mean plus S.E. and (*) indicates a significant dif [file 13578_2023_989_MOESM1_ESM.pdf]

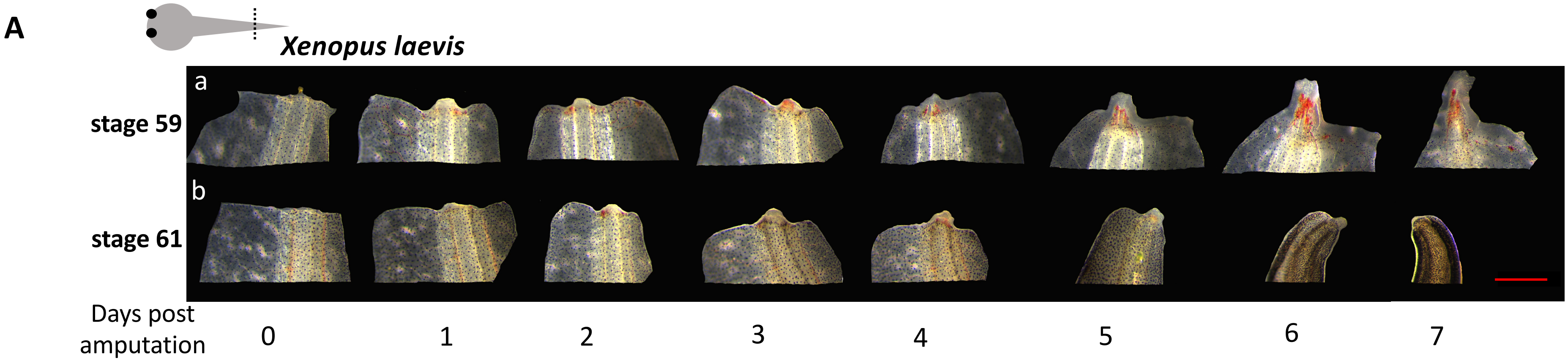

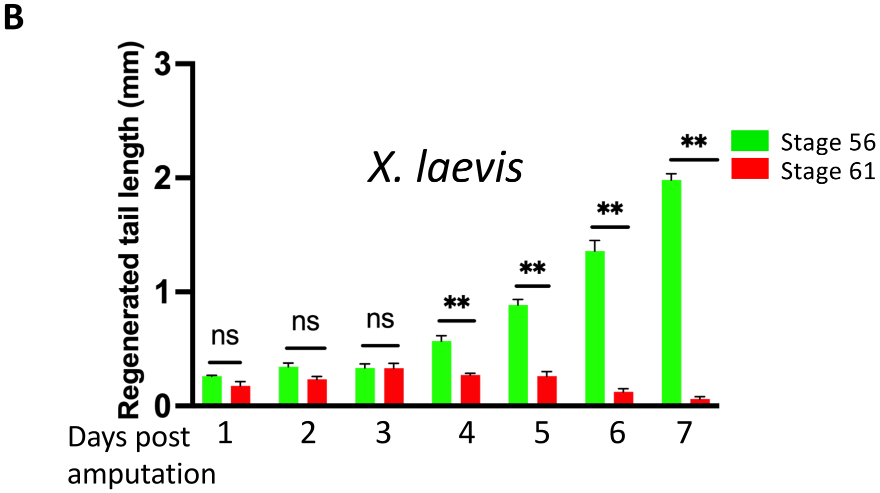

Figure S1. Tail loses regenerative ability during metamorphic climax in *Xenopus laevis*.

(A) Representative images of the tail at different time points after amputation for two tadpoles. The tadpole at stage 59, an early metamorphic climax stage, regenerated the tail completely by 7 days (a), while the one stage 61, a later stage when plasma T3 is around the peak level, failed to regenerate (b). Scale bar: 1mm.

(B) Quantitative analysis of the length of the regenerated tail after amputation at stage 56, an early metamorphic stage, or at stage 61. Note that regenerated tail gradually increased in length after amputation at stage 56, while the length of the regenerated tail at stage 61 appeared to decrease after 4 days, likely due to tail resorptions. The length of the regenerated portion of the tail was measured from at least 3 tadpoles at stage 56 or 61 and presented as mean ± SE. $**P < 0.01$, ns, not significant.

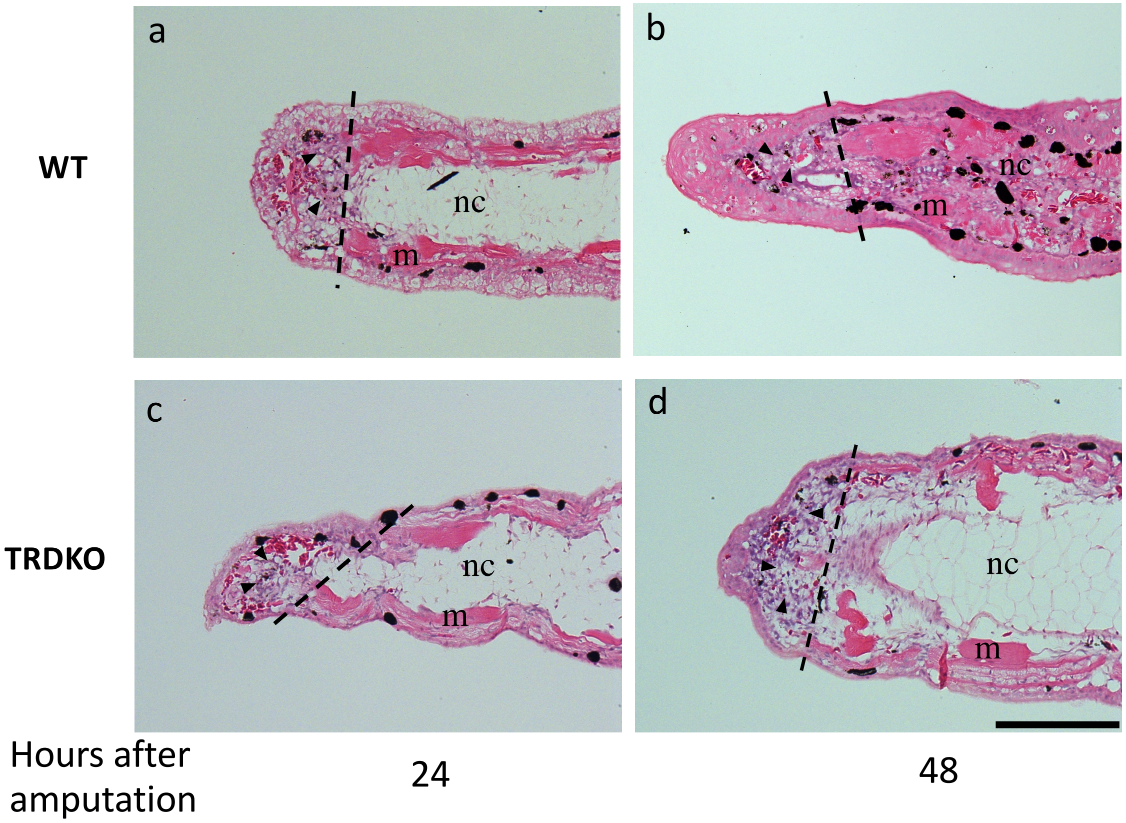

Figure s2. Tail can initiate regeneration in both wild type and TRDKO tadpoles at stage 61.

Frontal sections of wild type (a, b) and TRDKO (c, d) tadpole tail at 24 hours post-amputation (hpa) and 48 hpa that were stained with hematoxylin and eosin. Note that both wild type and TRDKO could complete wound healing and form special wound epidermis and blastema (as indicated in black arrowheads). Black dash lines indicate amputation site. nc, notochord; m, muscle. Scale bar: 150 µm.

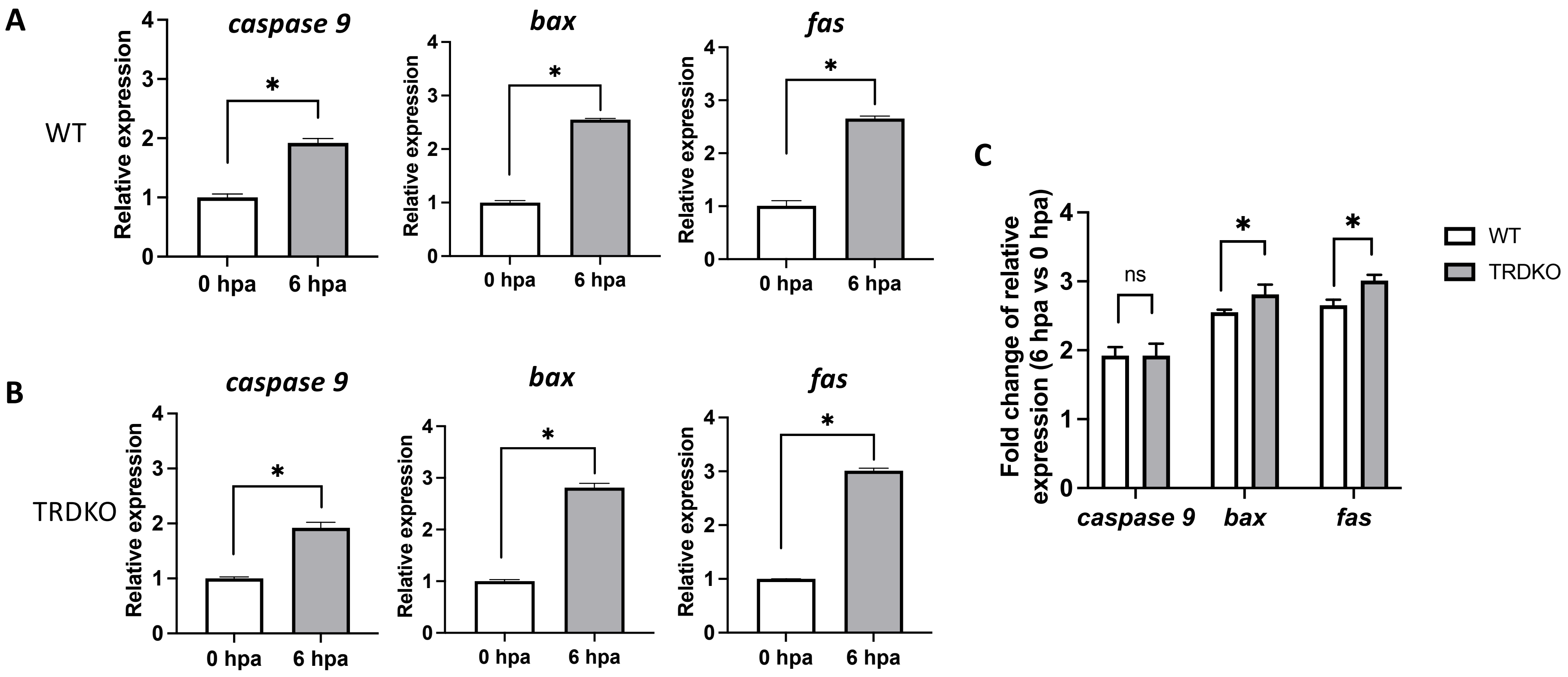

Figure S3. Analysis of the expression of apoptotic genes during wound healing in both wild-type (WT) and TRDKO animals by RT-qPCR.

The expression of three apoptotic genes (*caspase 9*, *bax,* and *fas*) at 0 hr and 6 hr after amputation in wild-type (A) and TRDKO tail (B). Each bar represents the mean plus S.E. and (*) indicates a significant difference between 6 hr and 0 hr ($P < 0.05$). (C) The ratio of the expression of the same three apoptotic genes at 6 hr to that at 0 hr for WT and TRDKO tail. Each bar represents the mean plus S.E. and (*) indicates a significant difference between the WT and TRDKO tail ($P<0.05$). ns indicates no significant difference. Note that the genes were induced during wound healing in both WT and TRDKO tadpoles, with TRDKO animals having a higher induction for two of the genes, consistent with the TUNEL staining results.

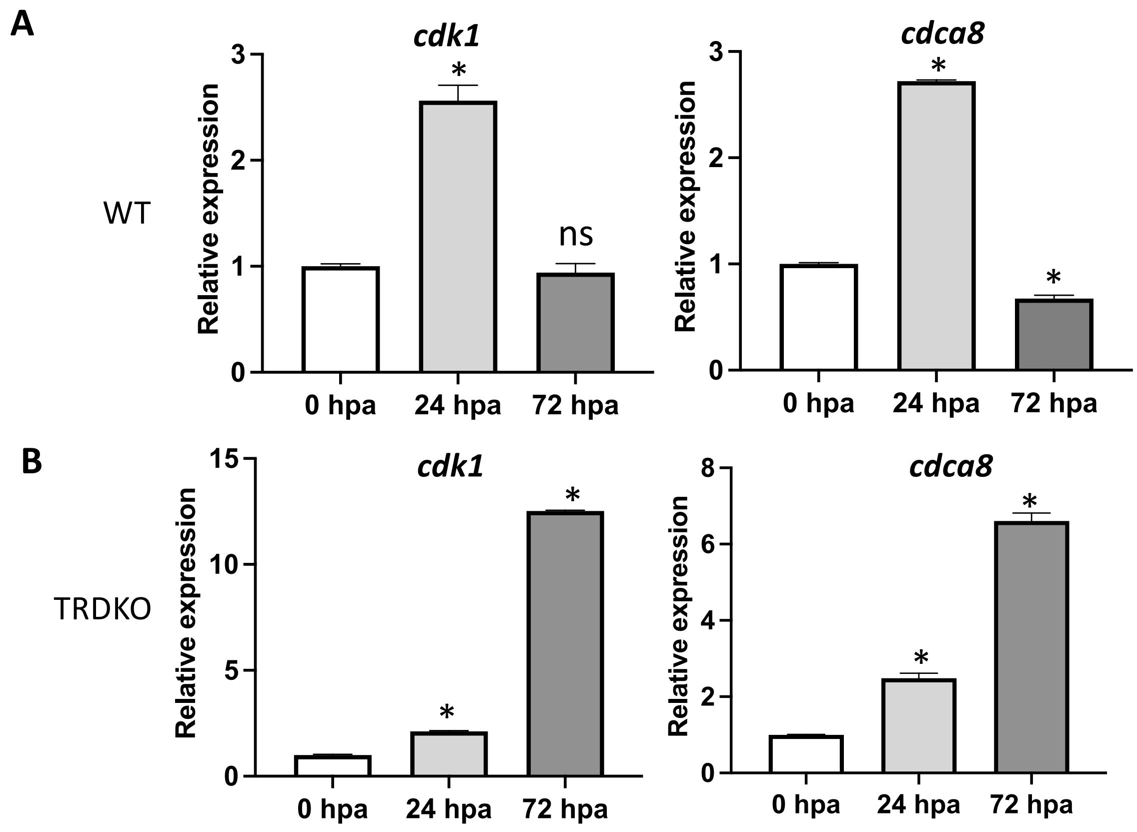

Figure S4. The regulation of cell cycle genes at patterning and outgrowth period during tail regeneration in both wild-type (WT) and TRDKO.

The expression of two known cell cycle genes (*cdk1* and *cdca8*) was analyzed by RT-PCR at 0 hr, 24 hr and 72 hr after amputation in wild-type (A) and TRDKO tail (B). Each bar represents the mean plus S.E. and (*) indicates a significant difference between 24 hr and 0 hr or 72 hr and 0 hr ($P < 0.05$). ns indicates no significant difference. Note that both genes were upregulated at 24 hr in both WT and TRDKO tadpoles. However, in WT tadpoles, their expression at 72 hr were returned to lower levels. These were consistent of the EdU staining results.

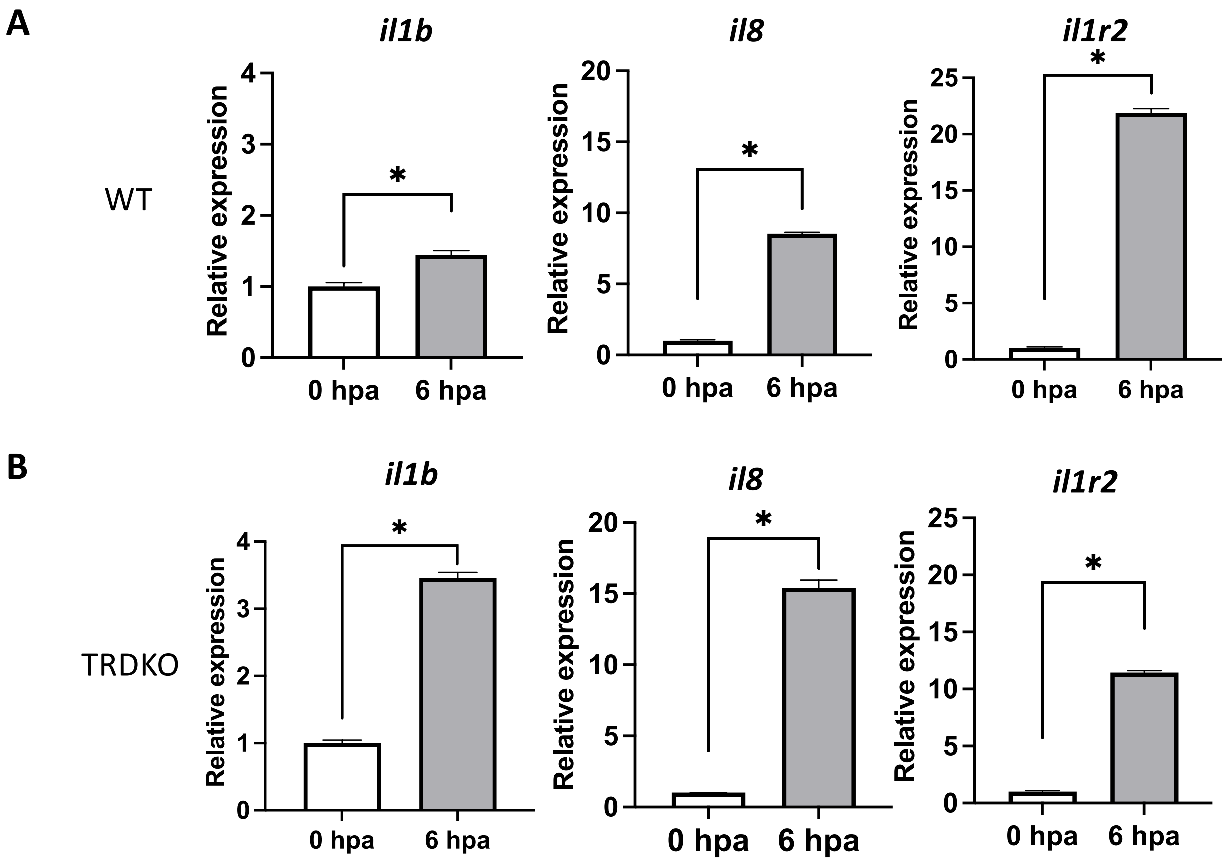

Figure S5. Analysis of the expression of inflammatory genes during wound healing in both wild-type (WT) and TRDKO animals by RT-qPCR.

The expression of three inflammatory genes (*il1b*, *il8* and *il1r2*) at 0 hr and 6 hr after amputation in wild-type (A) and TRDKO tail (B). Each bar represents the mean plus S.E. and (*) indicates a significant difference between 6 hr and 0 hr ($P < 0.05$). Note that the genes were induced during wound healing in both WT and TRDKO tadpoles.

Table S1. Primers used in RT-qPCR

Gene	Primer sequence (5'-3')		NCBI Reference No.
	Forward primer	Reverse primer	
rpl8	TTGCCAAGGTTGCTTTCCG	GTTTCAGGATTGTGGGAGATAACG	XM_012956636.2
caspase 9	GAGGAGTTTACGGAACAGATGG	AGTCACCGAACATCCTTTGTCT	NM_001123463.1
bax	TGCGGGAATATGTGGTCCAG	CAATGGTGGCAGTGAGGACA	NM_203854.1
fas	CACGGCCCATAGGACAAAGT	AACCCCCAACAACATCCGTT	NM_001102729.1
cdk1	GTTTGCTGGGAATCCAGCCT	TGTTGCTTTATGGCGACCCT	NM_203577.1
cdca8	TCCTTGGAAAGCGAAGGATTT	ACTGGCTGTCCACTTCTTTGAG	NM_001002902.1
il1b	GCAGAGACCACAGGCATTAGTATTAGC	GGAATGGAACTCAAGTCGGGAACC	NM_001015713.1
il8	GAGTGGAACCTGCTTGTGGT	GCCATGATTGCTGTTGGTTGT	XM_002942532.5
il1r2	TCTTTGGTGATGGCCGTGTA	GTCACAGTTCCTGCTCGTCTT	NM_001015713.1
mmp1	GGAACCAGTGAGGAGATGGC	ACTGTCCAGTATCCCCGTCA	NM_001030330.1
mmp13l	ATGCGTTGGGGTTGGATCAT	AGAGCCTGAATCCCTTGCAC	XM_002934925.4
mmp25	GAGCAAAGCCACACACAACTC	TCCACTCCTTTGCTGATGTCC	XM_031893750.1
mpo	AACAGACCCTGGACAACCAAC	ACCTGGCTGGCATCAACATAAG	XM_002935190.5
mmp7	CGCAATACAGAGGGCGTTTG	TAGAACTCCACTGGGGCCAT	NM_001005043.1
leptin	CAAAGATGTTGGCAAGGACCTT	GCTCATCTGGGATAAAATCCAAACCA	XM_002931835.4
cyp26a1	TGCCCTTCTTTGGAGAGACTCT	CGTACTTCCTTCGCTTAACTTGGA	XM_012966562.3
wnt3a	CAGCCATGAACAGGCACAAC	GATAGTCGCCGATCACCCTG	XM_002939308.5
wnt5a	ACTTGGGCTTCAGACCTGTG	GGGTTCATGGCTAACGACCA	XM_004914179.4
fgf10	CGGAGTAGGAAGCTCTTCTCCTACAC	TGTTAATGGCTTTCACTGCCACAA	NM_001016169.2
fgf8	ACAAGCCCAGGTAACTGTTCA	CTGGTAGGTTCGGATGAGCC	NM_001008162.1
